# Interaction between Dietary Vitamin D_3_ and Vitamin K_3_ in Gilthead Seabream Larvae (*Sparus aurata*) in Relation to Growth and Expression of Bone Development-Related Genes

**DOI:** 10.1155/2023/3061649

**Published:** 2023-05-23

**Authors:** U. Sivagurunathan, David Dominguez, Yiyen Tseng, María Jesús Zamorano, Antony Jesu Prabhu Philip, Marisol Izquierdo

**Affiliations:** ^1^Grupo de Investigación en Acuicultura (GIA), EcoAqua Institute, University of Las Palmas de Gran Canaria, Crta. Taliarte s/n, 35214 Telde, Spain; ^2^Feed and Nutrition Research Group, Institute of Marine Research, Bergen 5817, Norway

## Abstract

Vitamins D and K are essential fat-soluble nutrients that intervene in bone development processes among other biological functions. The present study is aimed at investigating the potential combined effect of dietary supplementation with vitamin D_3_ (cholecalciferol) and vitamin K_3_ (menadione) in gilthead seabream (*Sparus aurata*) larvae. For that purpose, seabream diets were supplemented with different combinations of vitamin D_3_/vitamin K_3_ (mg/kg diet) as follows: 0.00/0, 0.06/70, 0.06/170, 0.13/70, 0.13/170, 0.40/70, and 0.40/170. Feeding gilthead seabream larvae (22 days post hatch) for 21 days with the diets supplemented with 0.06-0.13 mg/kg vitamin D_3_ and 70 mg/kg vitamin K_3_ (diets 0.06/70 and 0.13/70) led to the highest larval growth and survival and the highest expression of important biomarkers of both bone development and health, such as *bmp2*, *osx*, and *mgp*, and calcium homeostasis, such as *pthrp* and *casr*. However, the increased supplementation with both vitamins at 0.40 mg/kg vitamin D_3_ and 170 mg/kg vitamin K_3_ (diet 0.40/170) reduced larval growth and survival, downregulated *bmp2* and *pthrp* expressions, and upregulated *osx* and *mgp*, causing an unbalance in the relative expression of these genes. The results of the present study have shown the interaction between vitamin D_3_ supplementation and vitamin K_3_ supplementation in larval performance and gene expression related to bone development and calcium homeostasis, denoting the significance of a correct balance between both vitamins in larval diets.

## 1. Introduction

Culturing marine fish larvae at commercial hatcheries is a demanding task, given their high sensitivity to biotic and abiotic factors [1]. Skeletal anomalies in larvae are more likely to develop during embryonic and postembryonic development, making it crucial to ensure proper nutrition for the larvae [2–5]. Micronutrients play a critical role in ensuring proper nutrition, but imbalances can lead to skeletal anomalies and affect larval performance during rearing [5–8]. Therefore, feed plays a significant role in larval performance, and the nutritional requirements for marine fish larvae differ from those of juveniles and broodstock [9, 10]. The larval stage is marked by numerous morphological and physiological changes that may affect nutrient requirements [11], which also vary across species [10, 12, 13]. Hatcheries typically feed larvae live prey, such as rotifers and Artemia, which have varying nutritional compositions and may lack some essential vitamins and minerals [11, 12, 14–16].

Among the different nutrients studied, fat-soluble vitamins D and K play crucial roles in the skeletal development of aquatic animals [17]. Vitamin D is vital for regulating plasma calcium and maintaining bone health, as well as for receptor-based biological functions in various tissues such as the gills, kidney, and intestine [18]. Vitamin K is essential for blood coagulation and the posttranslational modification of vitamin K-dependent proteins, which play critical roles in bone metabolism and growth control [19]. In juvenile fish studies, replacing fishmeal and fish oil with terrestrial ingredients in feed may decrease the dietary content of these vitamins, underscoring the need to establish their optimal dietary levels [20]. Several marine fish species have proposed individual dietary requirements for both vitamins, including Atlantic salmon (*Salmo salar*) postsmolt, Wuchang bream (*Megalobrama amblycephala*) fingerling, orange-spotted grouper (*Epinephelus coioides*) juveniles, and gilthead seabream (*Sparus aurata*) juveniles [18, 21–30]. However, quantitative requirements for larvae are still scarce, and only a few species, such as European seabass (*Dicentrarchus labrax*) and gilthead seabream for vitamin D and Senegalese sole (*Solea senegalensis*) for vitamin K, have had their optimal levels determined [31–33]. Additionally, live prey used in commercial hatcheries exhibit wide variations in their vitamin D and vitamin K contents [15], which could contribute to the high incidence of skeletal anomalies. Moreover, deficiencies or imbalances in either vitamin can lead to skeletal anomalies and affect the overall performance of the larvae during rearing.

Studies on humans or terrestrial animals demonstrate that vitamin D and vitamin K interact with each other to improve bone health and other biological functions [34–44]. However, this type of study is very scarce in fish [17], and none of them has been conducted during larval stages. The bone-forming cells (osteocytes) and bone-resorbing cells (osteoclasts) play significant roles in bone formation and remodeling in fish [19, 45, 46]. Proliferation and differentiation of these bone-forming cells are regulated by bone biomarkers [46–50]. The bone biomarkers analyzed in the present study and their role in regulating bone and calcium metabolism are listed in Table 1.

The bone-forming molecules work as a cascade during the bone development process and help in regulating other physiological pathways in fish [51]. The up- and downregulation of these biomarkers can be modulated by dietary nutrients [19, 20]. An abnormal expression of bone biomarkers causes severe problems in skeletal development and might lead to skeletal anomalies in cultured fish [52]. In marine fish species, the effect of dietary vitamin D and vitamin K on skeletal development has been studied individually by very few authors [20, 22, 31–33, 53–55]. Although the vitamin D and K interaction studies in fish species are scarce, a series of trials on Atlantic salmon studied the effects of dietary vitamin D and vitamin K on growth, bone minerals, and health performance [17, 56]. However, the combined effect of dietary vitamins D and K has not yet been studied in gilthead seabream larvae, despite its importance to understand the versatile functions of these vitamins in growth and skeletal development. Thus, considering that our previous studies determining the dietary vitamin D_3_ [32] and vitamin K_3_ [57] requirements in gilthead seabream larvae pave the way to study the interaction between both vitamins, the present study is aimed at understanding the potential interaction between vitamin D_3_ and vitamin K_3_ in gilthead seabream larvae concerning larval performance and expression of selected genes related to bone development and calcium regulation.

## 2. Materials and Methods

### 2.1. Larval Rearing

Natural spawns of gilthead seabream larvae were obtained from selected broodstock (PROGENSA (Spanish National Breeding Program) project [2] from GIA (Grupo de Investigación en Acuicultura, ECOAQUA Institute, Las Palmas de Gran Canaria University (ULPGC), Spain). Larvae (initial total length 6.27 ± 0.46 mm, dry body weight 0.22 ± 0.03 mg, mean ± SD) previously fed rotifers (*Brachionus plicatilis*) enriched with ORIGREEN (Skretting, Norway) until 22 days post hatch (dph) [58] were randomly distributed in 12 experimental tanks at a density of 1200 larvae in each tank. After the distribution, larvae were fed with experimental diets for 21 days (43 dph). All tanks (200 L, light grey color cylinder fiberglass tanks) were supplied with filtered seawater (36 g L^−1^ salinity) at an increasing rate of 0.3–1.0 L min^−1^ as a flow-through system along the feeding trials. Water entered the tank through a bottom mesh and was let out from the top to ensure water quality. Water was continuously aerated (125 mL min^−1^), attaining 5-8 g L^−1^ dissolved oxygen and saturation ranging between 60 and 80%. Water temperature (20 ± 2°C), photoperiod (12 h light : 12 h dark), and light intensity (1700 lux) were maintained constant throughout the experimental period (21 days).

### 2.2. Experimental Diets

Seven experimental microdiets of isoproteic (64.8 ± 0.56%) and isolipidic (20.48 ± 0.52%) contents were prepared containing four different levels of vitamin D_3_ (VD—cholecalciferol) (Sigma-Aldrich, CAS-67970) combined with two different levels of vitamin K_3_ (VK—menadione) (Sigma-Aldrich, CAS-58275) and one deficient diet (nonsupplemented) (Table 2). The chosen dietary vitamin levels were based on our previous study in gilthead seabream larvae fed different level of dietary vitamin D_3_ [32] and vitamin K_3_ [57]. The microdiets were prepared by grinding and sieving the ingredients below 125 microns. The ingredients were mixed in the following order: squid powder, water-soluble components, lipids, and fat-soluble vitamins, and finally, gelatin was mixed to obtain a homogenized mix. Then, the mix was pressed through a compress pelletizer (Severin, Suderm, Germany) and dried in the oven at 38°C for 24 h (Ako, Barcelona, Spain). After drying, pellets were grounded using a grinder (Braun, Kronberg, Germany) and sieved (Filtra, Barcelona, Spain) to achieve a pellet size of 250 and 500 *μ*m. Then, the microdiets were stored at refrigerated condition (2-8°C) throughout the experiment to avoid vitamin deterioration. Proximate analysis of the feed was conducted at GIA laboratories (Table 3). Each diet was tested in triplicates, and the diets were fed manually from 8.00 to 20.00 with 45-minute intervals for 21 days. Daily feed supply was increased from 3 g to 5 g along with the pellet size of 250–500 *μ*m, gradually during the experimental period. Larvae were periodically observed under stereoscope (Leica, M125, using Leica Application Suite software, Wetzlar, Germany) to determine the feed acceptance.

### 2.3. Larval Performance

Growth performance was determined at each sampling points at 7^th^, 14^th^, and 21^st^ days of feeding, by measuring total length and dry body weight. Total length of 20 larvae (anesthetized with clove oil) from each tank was measured by microscope (Leica microsystem, Leica Application Suite software, Germany). Whole-body weight was determined by triplicates of 10 starved larvae washed with distilled water and dried in a glass slide at an oven at 110°C for 24 h, followed by 1 h periods until constant weight was achieved. To evaluate the survival rate of larvae, the total live larvae from each treatment were collected and counted at the end of the experimental period.

### 2.4. Gene Expression Analysis

Larvae were collected for molecular studies at days 7 and 21 of the trial and preserved in 500 *μ*L RNALater (SIGMA, Madrid, Spain) and stored at -80°C. Total RNA was extracted from seabream larvae (approximately 100-150 mg per treatment) using the RNeasy Mini Kit (Qiagen, Hilden, Germany). Larvae were homogenized using TissueLyser-II (Qiagen, Germany) with QIAzol lysis reagent (Qiagen, Germany). Homogenized samples were centrifuged with chloroform for phase separation (12000 g, 15 min, 4°C). The upper aqueous phase was carefully pipetted into a tube containing 75% ethanol and mixed. This mix was transferred into an RNeasy spin column, where total RNA bonded to a membrane. RW1 and RPE buffers (Qiagen, Germany) were used to wash away contaminants. Finally, purified RNA was eluted with 30 *μ*L of RNase-free water. NanoDrop 1000 spectrophotometer (Thermo Scientific, Wilmington, DE, USA) was used to determine the quality and quantity of the eluted RNA. Synthesis of cDNA was conducted using the iScript cDNA Synthesis Kit (Bio-Rad, Hercules, CA, USA) according to manufacturer's instructions in an iCycler thermal cycler (Bio-Rad, USA). Primer efficiency was tested with serial dilutions of a cDNA pool (1, 1 : 10, 1 : 100, 1 : 200, and 1 : 1000). Real-time quantitative PCR was performed in an iQ5 Multicolor Real-Time PCR detection system (Bio-Rad, USA) using beta-actin (*β-actin*), ribosomal protein L27 (*rpl27*), and elongation factor 1 alpha 1 (*ef1a*) as the housekeeping genes in a final volume of 20 *μ*L per reaction well, and 100 ng of total RNA was reverse transcribed to complementary cDNA. The PCR conditions were the following: denaturation 95°C for 3 min and 30 s, followed by annealing at 40 cycles of 95°C for 15 s, 58.1-61°C for 30 s (Table 4), 72°C for 30 s, and 95°C for 1 min, and a final denaturation step from 58 to 95°C for 10 s. The nucleotides of the housekeeping and target gene primers are listed in Table 4.

### 2.5. Skeletal Anomalies

To determine the occurrence of skeletal anomalies, 150 larvae per treatment (i.e., 50 larvae per tank) were sampled at 43 dph and fixed in 4% formalin in phosphate buffer, pH 7.2, 0.1 M. Fixed larvae were stained for whole mount staining and examined under stereomicroscope (Leica M125, Wetzlar, Germany), and larval image (photographed using Leica DFC295 digital camera, Leica, Wetzlar, Germany) was processed using the Leica application suite (LAS 32167, Leica, Wetzlar, Germany). The stained larvae were examined for severe skeletal anomalies [5].

### 2.6. Data Analysis

All data were statistically analyzed using IBM SPSS Statistics v26.0. (IBM Corp., Chicago, IL, USA) and are expressed as means ± S.D. Data were treated for normality and homogeneity of variances using Levene's statistic, and means were compared to understand the statistical difference among the groups using Tukey's post hoc test (*p* < 0.05). To determine the effect of individual diet, one-way analysis of variance (ANOVA) was used, and for the interaction, two-way ANOVA was performed. SigmaPlot 12.3 (Systat Software, Inc., California, USA) was used to plot data in a three-dimensional mesh surface response plot to illustrate the interaction effect.

### 2.7. Ethical Statement

The study was conducted according to the European Union Directive (2010/63/EU) on the protection of animals for scientific purposes at Aquaculture Research Group (GIA) of ECOAQUA Institute, University of Las Palmas de Gran Canaria (ULPGC), Canary Islands, Spain. All experimentation performed at the ULPGC was approved by the Bioethical Committee of the University of Las Palmas de Gran Canaria (REF: 05/2021 CEBA ULPGC).

## 3. Results

### 3.1. Larval Performance

**Figure 1:**
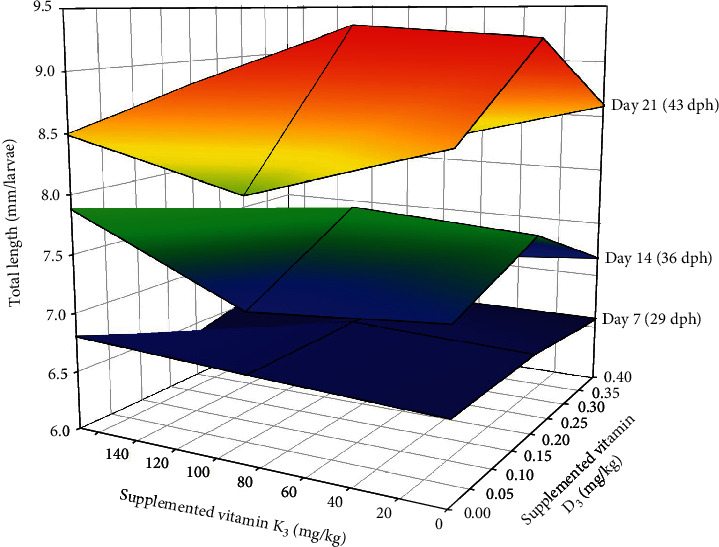
Effect of dietary vitamin D_3_ and vitamin K_3_ interaction on total length of gilthead seabream larvae at different sampling points (day 7: one-way ANOVA—*p* = 0.552 and two-way ANOVA—VD = 0.454, VK = 0.485, and VD^∗^VK = 0.826; day 14: one-way ANOVA—*p* = 0.061 and two-way ANOVA—VD = 0.925, VK = 0.551, VD^∗^VK = 0.315; day 21: one-way ANOVA—*p* = 0.03 and two-way ANOVA—VD = 0.001, VK = 0.536, and VD^∗^VK = 0.023).

Microscopic observation showed that all the experimental diets were well accepted by gilthead seabream larvae. After a period of 21-day feeding, the best survival was found in larvae fed 0.06/70 VD/VK diet and the lowest in those fed the 0.40/170 VD/VK diet (*p* = 0.08) (Table 5). The two-way ANOVA denoted a significant negative effect of increased dietary vitamin K_3_ levels on survival rates. Regarding the growth of larvae, there was no significant difference in total length on day 7 (29 dph) and day 14 (36 dph) (Figure 1). At the end of the trial, due to high mortality, no samples were recorded for length and weight in the group of larvae fed with high 0.40/170 VD/VK diet; hence, the highest growth in terms of total length was found in fish fed the 0.13/70 diet and the lowest in those fed the 0.40/70 diet (Table 5). Thus, increase in dietary vitamin D_3_ supplementation from 0.06 to 0.13 mg/kg significantly improved total length at the lower vitamin K_3_ supplementation level (70 mg/kg), but it did not at the higher supplementation level of vitamin K_3_ (170 mg/kg), denoting the interaction between both vitamins. Besides, further increase in dietary vitamin D_3_ from 0.13 to 0.40 mg/kg significantly reduced larval total length. Accordingly, the two-way ANOVA denoted that there was no significant effect on day 7 and day 14. But on day 21, there was a significant effect of dietary VD on total length, with an interaction between both vitamins. No significant effect was found on dry body weight, which is a less sensitive parameter at larval stages.

### 3.2. Bone Biomarker Gene Expression

**Figure 2:**
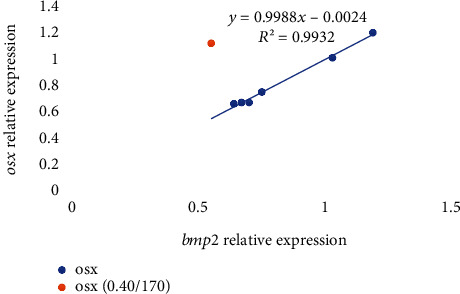
Relative expression of *osx* in relation to *bmp2* expression in gilthead seabream larvae fed diets supplemented with several levels of vitamin D_3_ and vitamin K_3_ for 21 days (*n* = 3, *p* < 0.05; blue dots for values of diets 0.00/0, 0.06/70, 0.06/170, 0.13/70, 0.13/170, and 0.40/70 and orange dot for 0.40/170).

**Figure 3:**
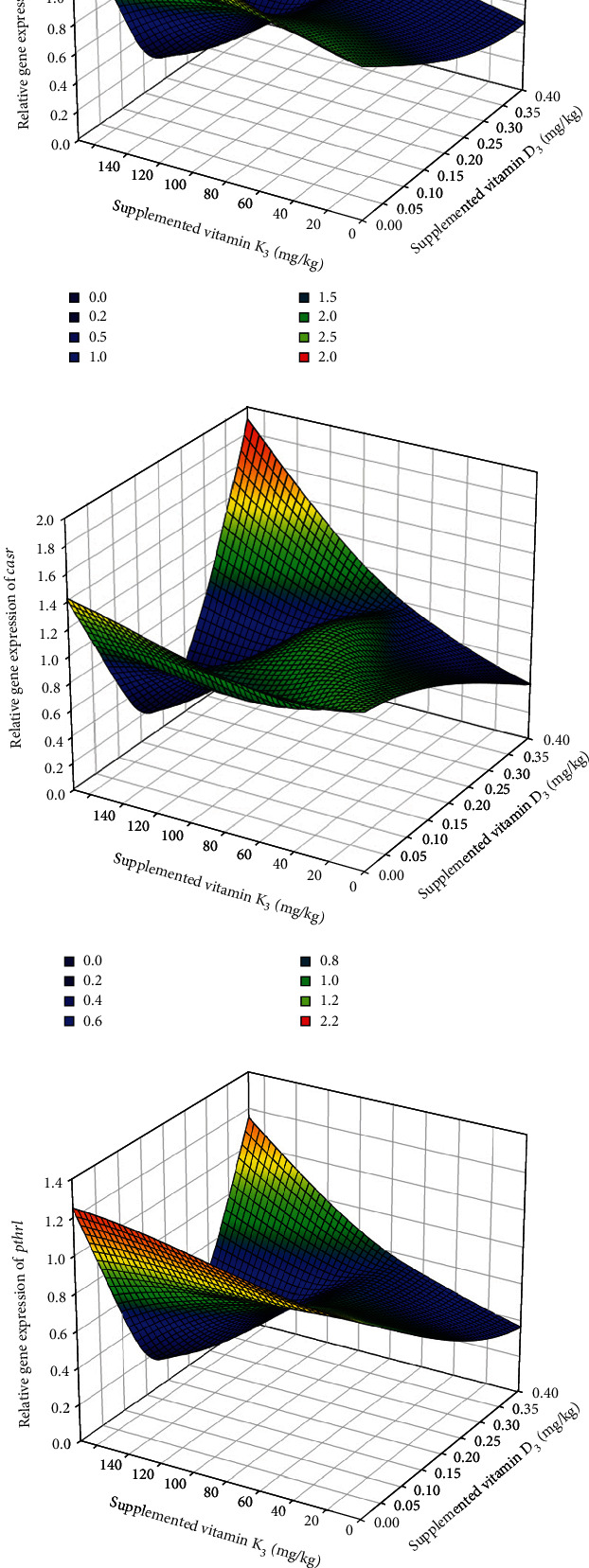
Effect of dietary vitamin D_3_ and vitamin K_3_ interaction on gene expression of vitamin K-dependent proteins (a) *oc* and (b) *mgp* and calcium regulators (c) *casr*, (d) *pthr1*, and (e) *pthrp* in gilthead seabream larvae at 29 dph.

**Figure 4:**
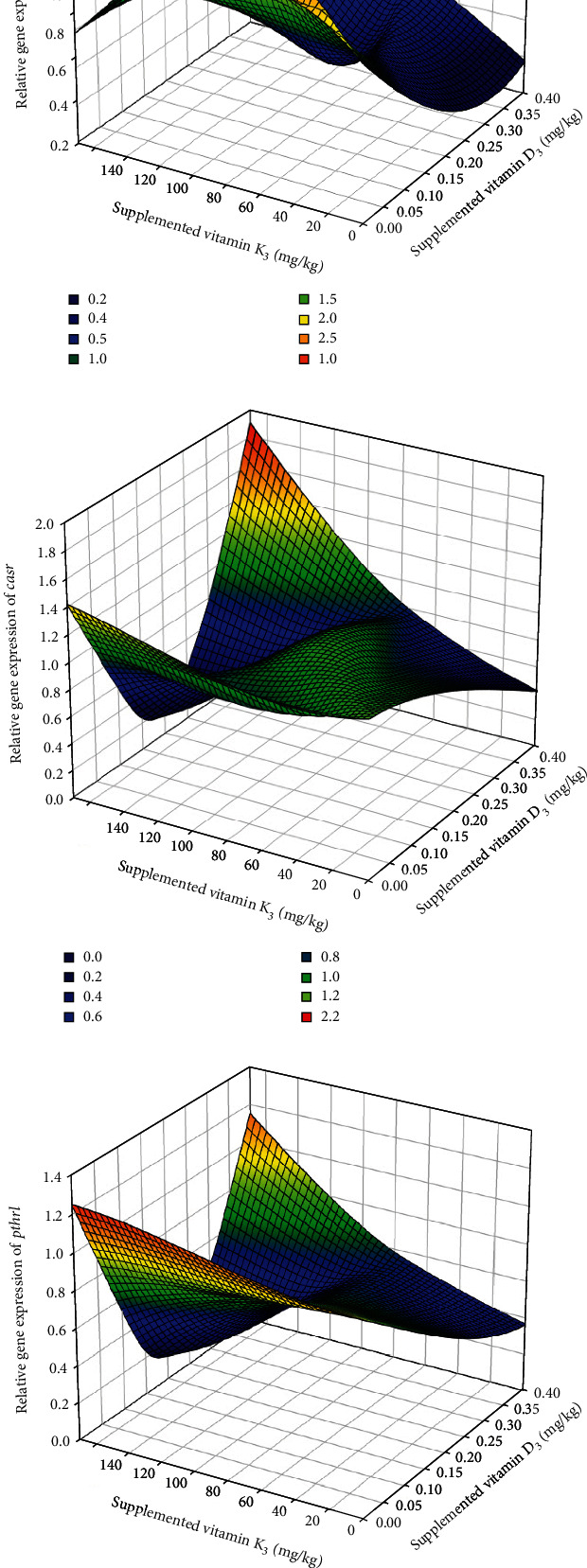
Effect of dietary vitamin D_3_ and vitamin K_3_ interaction on gene expression of vitamin K-dependent proteins (a) *oc* and (b) *mgp* and calcium regulators (c) *casr*, (d) *pthr1*, and (e) *pthrp* in gilthead seabream larvae at 43 dph.

**Figure 5:**
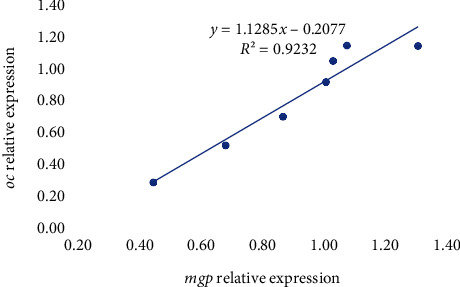
Relative expression of *oc* in relation to *mgp* expression in gilthead seabream larvae fed diets supplemented with several levels of vitamin D_3_ and vitamin K_3_ for 7 days (*n* = 3, *p* < 0.05; blue dots for values of diets 0.00/0, 0.06/70, 0.06/170, 0.13/70, 0.13/170 and 0.40/70 and orange dot for 0.40/170).

**Figure 6:**
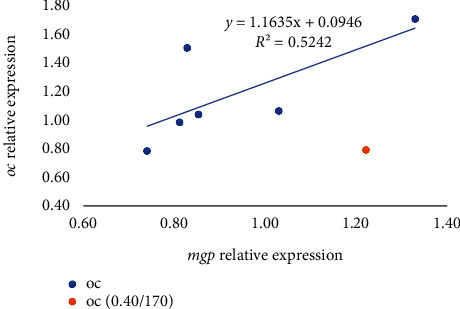
Relative expression of *oc* in relation to *mgp* expression in gilthead seabream larvae fed diets supplemented with several levels of vitamin D_3_ and vitamin K_3_ for 21 days (*n* = 3, *p* < 0.05; blue dots for values of diets 0.00/0, 0.06/70, 0.06/170, 0.13/70, 0.13/170 and 0.40/70 and orange dot for 0.40/170).

Among the genes related to bone metabolism, the relative gene expression of the bone morphogenic protein 2 (*bmp2*) was not affected by the diet after 7 days of feeding the experimental diets (Table 6) (*p* > 0.05), whereas at day 21, it was significantly upregulated in fish fed 0.06/70 diet in comparison to those fed the 0.40/170 diet. The *runx2* expression was not affected by the different dietary vitamin D_3_ and vitamin K_3_ levels tested neither at day 7 nor at day 21 (Table 6). The relative expression of osterix (*osx*) showed a similar expression pattern as *bmp2*, with no differences at day 7 (*p* < 0.05), but an upregulation at day 21 in fish fed 0.06/70 diet (Table 6). Moreover, in *osx* gene expression, a significant (*p* < 0.05) interaction between both vitamins was found at day 21 by the two-way ANOVA, denoting that although at the lowest vitamin D_3_ levels the increase in vitamin K_3_ downregulated *osx* (0.06/70 vs. 0.06/170), this effect was reversed at the highest vitamin D_3_ levels (0.40/70 vs. 0.40/170). Indeed, there was a very strong lineal regression (*R*^2^ = 0.99, *p* < 0.05; *y* = 0.9988*x*–0.0024) between *bmp2* and *osx* relative expressions at day 21 (Figure 2), except for the value at highest level of both vitamins (0.40/170 diet) that showed a higher expression in *osx* in relation to the *bmp2* expression. The expression of alkaline phosphatase (*alp*) was not significantly affected on day 7, although at day 21 there was a tendency for an upregulation at the highest vitamin D_3_ levels (0.40/70 and 0.40/170) (*p* = 0.054, Table 6). The osteocalcin (*oc*) expression was significantly downregulated in fish fed diet 0.13/170 at day 7 (*p* < 0.05), the two-way ANOVA suggesting an effect of vitamin D_3_ levels (*p* = 0.055, Table 6). At day 21, besides the mild vitamin D_3_ effect (*p* = 0.06), the two-way ANOVA also denoted the significant (*p* < 0.05) downregulatory effect of vitamin K_3_ increase (Table 6 and Figures 3(a) and 4(a)). After 7 days of feeding, the matrix-GLA protein (*mgp*) was significantly (*p* < 0.05) lowest in fish fed diet 0.13/170 and highest in fish fed 0.40/170 followed by those fed 0.40/70 (Table 6), and the two-way ANOVA analysis suggested the downregulatory effect of vitamin D_3_ increase (*p* = 0.056) and the interaction of both vitamins leading to an upregulation at the highest dietary levels of both vitamins (*p* = 0.067). At day 21, *mgp* expression followed a similar pattern at day 7, with the downregulation of this gene in fish fed the 0.40/70 diet followed by those fed intermediate levels of these vitamins (*p* < 0.05) and the highest in fish fed 0.06/70 followed by those fed 0.40/170 diet. The two-way ANOVA suggested the downregulatory effect of vitamin D_3_ (*p* = 0.054) and vitamin K_3_ (*p* = 0.059) and showed a significant interaction between both vitamins (*p* < 0.05, Figures 3(b) and 4(b)). Moreover, there was a strong lineal regression (*R*^2^ = 0.92, *p* < 0.05; *y* = 1.1285*x* − 0.2077) between *mgp* and *oc* relative expressions at day 7 (Figure 5). At day 21, a lineal regression (*R*^2^ = 0.5242, *y* = 1.1635*x* + 0.0946) was also observed between *mgp* and *oc* relative expressions (Figure 6), except for the value at highest level of both vitamins (0.40/170 diet) that showed a lower expression in *oc* in relation to the *mgp* expression.

**Figure 7:**
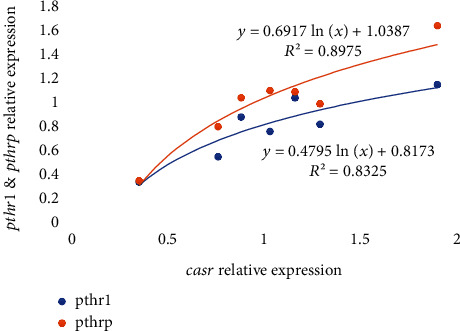
Relative expression of *pthr1* and *pthrp* in relation to *casr* expression in gilthead seabream larvae fed diets supplemented with several levels of vitamin D_3_ and vitamin K_3_ for 7 days (*n* = 3, *p* < 0.05; blue dots for *pthr1* values and orange dots for *pthrp*).

**Figure 8:**
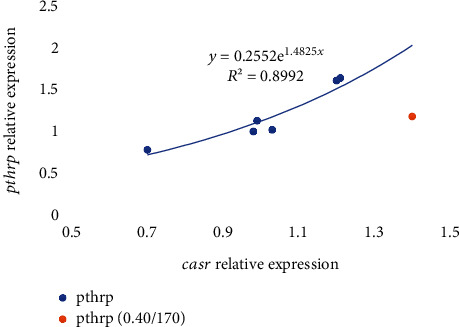
Relative expression of *pthrp* in relation to *casr* expression in gilthead seabream larvae fed diets supplemented with several levels of vitamin D_3_ and vitamin K_3_ for 21 days (*n* = 3, *p* < 0.05; blue dots for values of diets 0.00/0, 0.06/70, 0.06/170, 0.13/70, 0.13/170 and 0.40/70 and orange dot for 0.40/170).

Regarding the expression of other genes related to vitamin D and K metabolism, the calcium-sensing receptor (*casr*) expression at day 7 was significantly (*p* < 0.05) highest in fish fed 0.40/170 diet in comparison to those fed 0.13/170, whereas at day 21, no significant differences were found among the different dietary groups (Table 6 and Figures 3(c) and 4(c)). The expression of the parathyroid hormone receptor 1 (*pthr1*) and the parathyroid hormone-related protein (*pthrp*) at day 7 and increase in dietary vitamin K_3_ tend to downregulate these genes leading to the significantly (*p* < 0.05) lowest expression in fish fed the 0.13/170 diet, except in those fish fed the highest vitamin D_3_ levels, which showed the highest expression (Table 6 and Figures 3(d) and 3(e) and 4(d) and 4(e)). Moreover, there were strong logarithmic regressions between *casr* expression and *pthr1* (*R*^2^ = 0.8325, *y* = 0.4795ln(*x*) + 0.8173) or *pthrp* (*R*^2^ = 0.8975, *y* = 0.6917ln(*x*) + 1.0387) relative expressions at day 7 (Figure 7). After 21 days of feeding, increase in vitamin K_3_ downregulated both parathyroid hormone-related genes, which showed the lowest expression in fish fed 0.06/170 and 0.13/170 diets. In the case of *pthrp*, the two-way ANOVA also suggested (*p* = 0.053) the interaction between both vitamins, and, hence the increase in vitamin K_3_ in fish fed the highest levels of dietary vitamin D_3_ did not upregulate *pthrp* expression. There was also an exponential regression (*R*^2^ = 0.9162, *y* = 0.2552*e*1.4825*x*) between *casr* and *pthrp* expressions except for the value at highest level of both vitamins (0.40/170 diet) that showed a lower expression in *pthrp* in relation to the *casr* expression (Figure 8). However, the comparison between expression of bone biomarker and calcium regulators did not show any significant changes with respect to different sampling points between different dietary groups (Table 7).

### 3.3. Skeletal Studies

**Figure 9:**
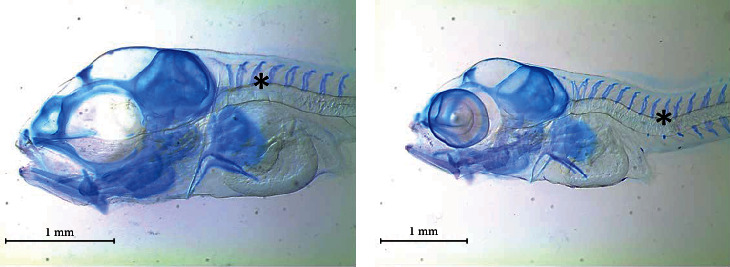
Skeletal anomalies found in gilthead seabream larvae fed diets supplemented with several levels of vitamin D_3_ and vitamin K_3_ for 21 days: (a) abdominal kyphosis and (b) haemal lordosis.

Supplementation with different combinations of dietary VD and VK showed no significant difference in frequency of skeletal anomalies (Figure 9). The occurrence of abdominal kyphosis and haemal lordosis (Table 8) did not differ among the different dietary treatments. Similarly, two-way ANOVA also showed no effect on skeletal anomalies.

## 4. Discussion

Optimum dietary levels of either vitamin D or vitamin K are necessary for larval performance and skeletal development in marine fish larvae [31, 32]. Despite the clear interaction between these vitamins in mammals [42], studies in fish are very scarce and none were conducted during larval stages [56, 59]. The present study showed the significant interaction between both vitamins in larval performance and gene expression related to bone development, denoting the importance of a correct balance between both vitamins in larval diets.

Regarding larval performance, neither growth nor survival was significantly reduced in fish fed diets without supplementation with vitamin D_3_ or vitamin K_3_. However, an increase in both vitamins led to the best larval performance, suggesting that the basal dietary levels were only marginally deficient. Thus, the increase in dietary vitamin D_3_ supplementation from 0.06 to 0.13 mg/kg improved the total length of larvae fed the lower dietary vitamin K_3_ supplementation level (70 mg/kg). This agrees well with the growth-promoting effect of vitamin D observed in mammals [60] and fish [25, 27, 61], particularly during early developmental stages [31]. In contrast with the present results, in gilthead seabream juveniles, increased supplementation of vitamin D_3_ had only a mild effect on growth improvement [20], confirming the stronger growth-promoting effect and importance of this vitamin in early stages. However, increase in vitamin D_3_ from 0.06 to 0.13 mg/kg did not improve growth at the higher vitamin K_3_ supplementation level (170 mg/kg), denoting a clear interaction between both vitamins confirmed by the 2-way ANOVA statistical analysis. The present study is the first one that shows the interaction effect on the larval growth. On the contrary, in the present study, the increase in vitamin K_3_ supplementation level from 70 or 170 mg/kg did not affect seabream growth, in agreement with the lack of effect of vitamin K supplementation on growth of salmon fry [62].

In the present study, the further increase in the dietary vitamin D_3_ up to 0.4 mg/kg significantly reduced the larval growth, denoting the negative effects of excessive dietary vitamin D_3_, and this conclusion is in agreement with the previous studies on seabream [32] and other species [18]. In turbot (*Psetta maxima*), the overdose of vitamin D_3_ produced an intestinal inflammation and reduced the gut microbiota diversity [63] that could be related with an impairment of growth. Despite the increase in vitamin K_3_ supplementation from 70 to 170 mg/kg did not affect seabream growth, at the highest vitamin D_3_ and K_3_ supplementation levels, the survival rate was significantly reduced. Poor survival rates may be attributed to the toxic effect of vitamin overdose, where the larvae fed 0.4 mg/kg vitamin D_3_ with 70 mg/kg showed higher survival than larvae fed 170 mg/kg vitamin K_3_. This denotes that the highest dietary vitamin D_3_ content accompanied with the lower vitamin K_3_ level had better effect on survival than in larvae fed on the higher vitamin K_3_ content. This in agreement with the authors' previous study, where the larvae were fed on diets supplemented with vitamin D_3_ at 0.50 mg/kg and vitamin K_3_ at 173 mg/kg supplementation levels that reduced the larval survival rate [32]. Further studies in mammals and other species showed that the excess menadione supplementation would impair the growth and survival rate [23] and increase the binding capacity of vitamin D transporter to vitamin D, which results in increased vitamin D receptor activity. The vitamin D receptor participates in the pathophysiological process by enhancing the metabolite effect in larvae [64, 65].

The highest larval survival rate was recorded in larvae fed the diet supplemented with 0.06 and 70 mg/kg vitamin D_3_ and vitamin K_3_, respectively. These larvae also showed, at the end of the trial, the highest expression of *bmp2*, *osx*, and *mgp*, which are important biomarkers of bone development and health. The *bmp2* is an osteochondrogenic factor which initiates bone formation and bone healing, inducing the expression of other *bmp*s, whereas *osx* regulates late osteogenesis and bone matrix mineralization [66, 67]. In the present study, *osx* expression followed a linear relation with *bmp2* expression, in larvae fed the diet supplemented with 0.06 and 70 mg/kg vitamin D_3_ and vitamin K_3_, respectively, showing the highest values for both genes. The *mgp* promotes osteoblast proliferation and bone formation through the Wnt/*β*-Catenin signaling pathway [68], affecting mineralization and osteoclast differentiation [69]. The expression of *mgp* occurs during early development of gilthead seabream [70]. In the present study, *mgp* expression was associated with increased *oc* expression, with the highest values for both genes found in the larvae fed the diet supplemented with 0.06 and 70 mg/kg vitamin D_3_ and vitamin K_3_, respectively. Osteocalcin is produced by osteoblasts and incorporated into the bone matrix, but it is also involved in energy metabolism and other metabolic functions [71]. Vitamin K plays an important role in the carboxylation of osteocalcin and its posterior binding to hydroxyapatite [17]. Besides, both vitamins regulate *oc* expression [72, 73]. The highest expression of *mgp* and its correlation with *oc* in larvae fed the diet supplemented with 0.06 mg/kg vitamin D_3_ and 70 mg/kg vitamin K_3_ agree well with the combined effect of vitamin D and vitamin K on osteoblast activity and osteoclast formation, upregulation of osteoblastic genes, and bone health improvement found in other species [43, 74]. Besides, vitamin D enhances the vitamin K-dependent proteins to induce bone formation along with vitamin K [38]. Thus, in mammals, the combination of both vitamins is more effective in the prevention of bone loss and fracture risk [42, 75].

However, further elevation of vitamin K_3_ supplementation from 70 to 170 mg/kg at 0.06 mg/kg vitamin D_3_ supplementation downregulated *bmp2*, *osx*, and *mgp*, denoting the negative effect of increased vitamin K_3_. These results agree well with the indirect effect of vitamin K on *bmp2* production translating into MK-7 [76]. Besides, vitamin K_3_ intervenes in the activation by carboxylation of the matrix Gla protein (*mgp*), which is a main inhibitor of *mgp* expression. Moreover, the combined higher concentrations of both dietary vitamin D_3_ and K_3_ (0.40 and 170 mg/kg, respectively) further downregulated *bmp2* expression and upregulated *osx* and *mgp*, causing an imbalance in the expression of *osx* in relation to *bmp2* and in the expression of *oc* in relation to *mgp*. These results reflected the interaction of both vitamins denoted by the two-way ANOVA and agree well with the downregulation of *bmp2* found in fish larvae fed high doses of vitamin D_3_ [31, 32]. Indeed, vitamin D is actively involved in the regulation of *bmp2* and *osx* expression [77]. Whereas *bmp2* is an osteochondrogenic factor, *osx* inhibits chondrogenesis, and the imbalance in the expression of both genes could be responsible for an impairment in bone formation, particularly in endochondral bones. This agrees well with the high incidence of cranial anomalies found in gilthead seabream larvae fed excessive levels of vitamin D_3_ [32].

There is much evidence that, particularly during early developmental stages, both vitamin D and K are necessary for the formation of strong bones and the prevention of skeletal anomalies [38, 53, 78]. Besides, feeding gilthead seabream with high levels of vitamin D_3_ leads to cranial malformations [32], whereas excessive levels of vitamin K_3_ cause a high incidence of kyphosis [57]. In the present study, despite the imbalance found in the relative expression of bone health related genes and their significant differences among larvae fed different vitamin supplementation levels, no significant differences were found in the incidence of skeletal anomalies. This lack of evidence on the skeletal anomalies could be attributed to the sufficient contents of vitamins D_3_ and K_3_ in the diet without supplementation of these vitamins, preventing the marked deficiency symptoms such as poor growth or high incidence of bone anomalies. On the other hand, the highest supplementation levels of vitamins D_3_ and K_3_ (0.40/170 mg/kg) caused an imbalance in the expression of some bone biomarkers, significantly reduced larval survival rate (7.92% vs. 28.39%), and induced the weakest and deformed larvae. Moreover, the supplementation levels of vitamins D_3_ and K_3_ tested in the present study were lower than those inducing skeletal anomalies in the previous studies [57]. Nevertheless, further studies should be conducted to find out the potential interaction between these two vitamins in relation to the occurrence of skeletal anomalies.

After 21 days of feeding the experimental diets, the expression of *pthrp* followed an exponential regression with *casr* expression, with the highest values found in larvae fed the diets 0.06/70 and 0.13/70. These results agree well with the highest survival and growth found in these larvae and with the upregulation of *pthrp* found in juveniles of seabream when dietary vitamin D is increased over the deficient levels [79]. Besides, the results also agree with the strong correlation between *pthrp* and *casr* found in mammals [80]. The parathyroid hormone-related protein (*pthrp*) is related to the function of PTH and among other roles regulates endochondral bone development. The calcium-sensing receptor (CaSR) is a class C G-protein coupled receptor, sensitive to extracellular levels of calcium. It is expressed in the parathyroid gland, among other organs, and calcium inhibits parathyroid hormone (PTH) release. Both factors are the main regulators of calcium homeostasis [81], and their ratios are determinant for the correct balance of intra- and extracellular calcium.

However, the increase in the dietary vitamin K_3_ supplementation level from 70 to 170 mg/kg at the lowest dietary vitamin D_3_ supplementary level downregulated *pthrp*. Moreover, the highest supplementation level of both vitamins up to 0.40 vitamin D_3_ mg/kg and 170 mg/kg vitamin K_3_ markedly reduced the expression of *pthrp* in relation to *cars* expression. This unbalance in the expression of these two factors, which are important for calcium homeostasis, could be responsible for an excessive elevation of plasma calcium concentrations that could in turn inhibit *pthrp* release [80].

## 5. Conclusions

The present study showed the interaction between vitamin D_3_ supplementation and vitamin K_3_ supplementation in larval performance and gene expression related to bone development and calcium homeostasis, denoting the significance of a correct balance between both vitamins in larval diets. Supplementation with 0.06-0.13 mg/kg vitamin D_3_ and 70 mg/kg vitamin K_3_ led to the highest larval growth and survival and the highest expression of important biomarkers of both bone development and health, such as *bmp2*, *osx*, and *mgp*, and calcium homeostasis, such as *pthrp* and *casr*. However, the combined increase in the supplementation of both vitamins up to 0.40 mg/kg vitamin D_3_ and 170 mg/kg vitamin K_3_ reduced the larval growth and survival, downregulated *bmp2* and *pthrp* expression, and upregulated *osx* and *mgp*, causing an unbalance in the relative expression of these genes.

## Figures and Tables

**Table 1:** List of bone biomarkers and their function on bone development and calcium metabolism.

Bone biomarkers	Function	Reference
Bone morphogenic protein 2 (*bmp2*)	Bone cell differentiation	[20, 30, 31, 47, 50–52, 82–85]
	Development of cartilage and bone	
	Transforming growth factor beta (TGF-*β*) signaling pathway	
RUNX family transcription factor 2 (*runx2*)	Osteoblast differentiation	
	Development of cartilage and bone	
Osterix (*osx)*	Bone formation and remodeling	
	Activates gene cascade during differentiation of preosteoblast to mature osteoblast	
Alkaline phosphatase (*alp*)	Bone mineralization	
	Reduces the extracellular pyrophosphate concentration	
Osteocalcin (*oc*)	Noncollagenous bone protein synthesized in the extracellular matrix of osteoblast	
	Contributes to bone mineralization by promoting calcium deposition	
Matrix Gla protein *(mgp)*	Inhibits calcification	
	Regulates osteoclastogenesis	
Calcium-sensing receptor (*casr)*	Regulates calcium homeostasis	
	Can act directly on bone cells to induce bone modeling or remodeling	
Parathyroid hormone 1 receptor *(pthr1)*	Modifies the gene involved in mineralization	
Parathyroid hormone-related protein *(pthrp)*	Hypercalcemic hormone	
	Calcium homeostasis	

**Table 2:** Supplemented levels of VD_3_ and VK_3_ in the experimental diets (mg/kg).

Diet	0	0.06/70	0.06/170	0.13/70	0.13/170	0.40/70	0.40/170
VD_3_ (mg/kg)	0	0.06	0.06	0.13	0.13	0.40	0.40
VK_3_ (mg/kg)	0	70	170	70	170	70	170

**Table 3:** Ingredients (g/kg diet) and analyzed proximate composition (%) of the experimental diets supplemented with different levels of vitamin D_3_ and vitamin K_3_.

Experimental diet							
Diet	0	0.06/70	0.06/170	0.13/70	0.13/170	0.40/70	0.40/170
Ingredients (%)							
Squid powder^∗^	70.2	70.2	70.2	70.2	70.2	70.2	70.2
Gelatin	3	3	3	3	3	3	3
Krill oil^†^	13	13	13	13	13	13	13
Mineral premix^˟^	4.5	4.5	4.5	4.5	4.5	4.5	4.5
Sel-Plex	0.3	0.3	0.3	0.3	0.3	0.3	0.3
Vitamin premix^˟^	6	6	6	6	6	6	6
Attractants^ᵜ^	3	3	3	3	3	3	3
Proximate analysis (% wet weight)							
Moisture	8.2 ± 0.16	6.79 ± 0.01	7.47 ± 0.04	7.47 ± 0.16	7.99 ± 0.09	8.9 ± 0.04	7.86 ± 0.03
Ash	7.44 ± 0.03	7.59 ± 0.02	7.51 ± 0.02	7.49 ± 0.04	7.48 ± 0.02	7.39 ± 0.01	7.07 ± 0.49
Lipid	22.03 ± 0.96	20.36 ± 0.33	20.09 ± 0.38	20.25 ± 0.01	20.63 ± 0.58	19.46 ± 0.54	19.83 ± 0.83
Protein	63.72 ± 0.76	65.33 ± 0.28	63.9 ± 1.85	65.46 ± 0.52	64.71 ± 0.4	64.88 ± 0.02	65.34 ± 0.06

^∗^Bacarel Express, code: 70400, United Kingdom. ^†^Krill, high phospholipids, Aker BioMarine, Fjordalléen, Norway. ^˟^Vitamin and mineral premix used according to [86] with modifications: vitamin premix (mg/100 g): water-soluble vitamins (cyanocobalamin: 0.03, astaxanthin: 5, folic acid: 5.44, pyridoxine-HCl: 17.28, thiamine-HCl: 21.77, riboflavin: 72.53, calcium pantothenate: 101.59, P-aminobenzoic acid: 145, nicotinic acid: 290.16, and inositol: 1450.9) and fat-soluble vitamins (retinol acetate: 0.24, alpha-tocopherol acetate: 150, and vitamin C: 180). Mineral premix (mg/100 g): sodium chloride (NaCl): 215.133, magnesium sulfate heptahydrate (MgSO4.7H2O): 677.545, sodium dihydrogen phosphate monohydrate (NaH2PO4.H2O): 381.453, dipotassium hydrogen phosphate (K2HPO4): 758.949, calcium dihydrogen phosphate dihydrate (Ca(H2PO4).2H2O): 671.61, ferric citrate (FeC6H5O7): 146.884, calcium lactate (C3H5O3.1/2Ca): 1617.21, aluminum sulfate hexahydrate (Al2(SO4)3.6H2O): 0.693, zinc sulfate heptahydrate (ZnSO4.7H2O): 14.837, copper sulfate pentahydrate (CuSO4.5H2O): 1.247, manganese sulfate monohydrate (MnSO4.H2O): 2.998, potassium iodide (KI): 0.742, and cobalt sulfate heptahydrate (CoSO4.7H2O): 10.706. ^ᵜ^Attractants were used based on [87].

**Table 4:** Sequences of primers used in gene expression studies.

Gene	Nucleotide sequence (5′-3′)	Annealing temperature	Accession number
Beta-actin (*β-actin*)	F: TCTGTCTGGATCGGAGGCTC	58.1	X89920
	R: AAGCATTTGCGGTGGACG		
Elongation factor 1-alpha (*ef1α*)	F: CTTCAACGCTCAGGTCATCAT	60	AF184170
	R: GCACAGCGAAACGACCAAGGGGA		
Ribosomal protein L27 (*rpl27*)	F: AAGAGGAACACAACTCACTGCCCCAC	68	AY188520
	R: GCTTGCCTTTGCCCAGAACTTTGTAG		
Bone morphogenic protein 2 (*bmp2*)	F: GTGGCTTCCATCGTATCAACATTTT	60	JF261172.1
	R: GCTCCCCGCCATGAGT		
RUNX family transcription factor 2 (*runx2*)	F: GCCTGTCGCCTTTAAGGTGGTTGC	61	AJ619023
	R: TCGTCGTTGCCCGCCATAGCTG		
Osterix (*osx)*	F: CAGTCAGGGATTCAGCAACA	60	ERR22591_isotig06993
	R: GGTGAAGGAGCCAGTGTAGG		
Alkaline phosphatase (*alp*)	F: AGAACGCCCTGACGCTGCAA	61	AY266359
	R: TTCAGTATACGAGCAGCCGTCAC		
Osteocalcin (*oc*)	F: GGCAGCCATCTGTCTGACTT	58.1	AF048703
	R: GGTCCGTAGTAGGCCGTGTA		
Matrix Gla protein *(mgp)*	F: CGCCCGAAATACACCTCAGA	60	AY065652
	R: GACGGACGGATACTAGGAGTCTA		
Calcium-sensing receptor (*casr)*	F: GCTTCTCCAGCTCGCTCATC	60	AJ289717
	R: AGGCGGGCTGGCGTAA		
Parathyroid hormone 1 receptor *(pthr1)*	F: GAACCTGCCCGGCTACGTGAAG	60	AJ619024
	R: GCTCCTGTCCCG ACGAGGGTAT		
Parathyroid hormone-related protein *(pthrp)*	F: GAGGCAAATGAATGGAACAG	60	AF197904
	R: TGGCCAGCTCAAAACTTGT		

**Table 5:** Growth performance of gilthead seabream larvae fed diets supplemented with several levels of vitamin D_3_ and vitamin K_3_ for 21 days.

	0	0.06/70	0.06/170	0.13/70	0.13/170	0.40/70	0.40/170	One-way ANOVA(*p* value)	Two-way ANOVA(*p* value)		
									VD	VK	VD^∗^VK
Survival (%)	19.03 ± 5.04^ab^	28.39 ± 4.38^b^	19.81 ± 5.96^ab^	22.92 ± 11.95^ab^	19.53 ± 9.83^ab^	24.56 ± 5.14^ab^	7.92 ± 0.00^a^	0.08	0.183	0.012	0.291
Total length (mm/larvae)	8.58 ± 0.25^ab^	8.20 ± 0.16^a^	8.42 ± 0.34^ab^	9.06 ± 0.05^b^	8.74 ± 0.22^ab^	8.24 ± 0.40^a^	_	0.03	0.001	0.536	0.023
Body weight (mg/larvae)	0.54 ± 0.02	0.52 ± 0.02	0.52 ± 0.07	0.55 ± 0.05	0.49 ± 0.13	0.44 ± 0.12	_	0.64	0.316	0.604	0.581

^∗^Different letters in a row denote significant differences between groups fed different diets (mean ± SD, *n* = 3, *p* < 0.05).

**Table 6:** Relative gene expression of bone biomarkers and calcium regulators in gilthead seabream larvae fed diets supplemented with several levels of vitamin D_3_ and vitamin K_3_ for 21 days.

	Diet								One-way ANOVA (*p* value)	Two-way ANOVA (*p* value)		
	Gene	0	0.06/70	0.06/170	0.13/70	0.13/170	0.40/70	0.40/170		VD	VK	VD^∗^VK
Day 7 (29 dph)	*bmp2*	1.08 ± 0.40	1.13 ± 1.11	1.09 ± 0.62	0.96 ± 0.64	0.42 ± 0.15	1.11 ± 0.79	0.79 ± 0.33	0.659	0.751	0.267	0.48
	*runx2*	1.04 ± 0.31	0.77 ± 0.44	1.27 ± 0.53	1.16 ± 0.72	1.04 ± 0.55	1.14 ± 0.56	1.12 ± 0.56	0.892	0.473	0.592	0.185
	*osx*	1.06 ± 0.41	0.83 ± 0.14	1.24 ± 0.71	0.78 ± 0.21	0.80 ± 0.45	0.67 ± 0.08	1.53 ± 1.19	0.201	0.189	0.859	0.164
	*alp*	1.00 ± 0.10	1.15 ± 0.07	1.74 ± 0.71	1.34 ± 0.36	1.09 ± 0.22	1.16 ± 0.51	1.31 ± 0.44	0.102	0.674	0.459	0.371
	*oc*	1.05 ± 0.32^b^	0.92 ± 0.57^b^	1.15 ± 0.47^b^	0.70 ± 0.48^ab^	0.29 ± 0.07^a^	0.52 ± 0.15^ab^	1.14 ± 0.72^b^	0.03	0.055	0.547	0.344
	*mgp*	1.03 ± 0.24^ab^	1.01 ± 0.28^ab^	1.07 ± 0.32^ab^	0.87 ± 0.31^ab^	0.44 ± 0.12^a^	0.68 ± 0.27^ab^	1.31 ± 0.57^b^	0.009	0.056	0.297	0.067
	*casr*	1.16 ± 0.66^ab^	0.88 ± 0.32^ab^	0.92 ± 0.60^ab^	1.03 ± 0.65^ab^	0.35 ± 0.06^a^	0.76 ± 0.14^ab^	1.90 ± 1.30^b^	0.054	0.104	0.501	0.015
	*pthr1*	1.04 ± 0.29^ab^	0.88 ± 0.45^ab^	0.82 ± 0.49^ab^	0.76 ± 0.38^ab^	0.34 ± 0.02^a^	0.55 ± 0.16^ab^	1.15 ± 0.53^b^	0.035	0.488	0.457	0.113
	*pthrp*	1.09 ± 0.45^ab^	1.04 ± 0.69^ab^	0.99 ± 0.51^ab^	1.10 ± 0.84^ab^	0.35 ± 0.05^a^	0.80 ± 0.23^ab^	1.97 ± 0.95^b^	0.035	0.068	0.595	0.005

Day 21 (43 dph)	*bmp2*	1.03 ± 0.29^ab^	1.19 ± 0.47^b^	0.70 ± 0.30^ab^	0.67 ± 0.31^ab^	0.75 ± 0.33^ab^	0.64 ± 0.26^ab^	0.55 ± 0.26^a^	0.026	0.178	0.352	0.51
	*runx2*	1.02 ± 0.25	0.93 ± 0.36	0.80 ± 0.43	1.02 ± 0.31	1.02 ± 0.70	1.35 ± 0.90	0.95 ± 0.29	0.705	0.746	0.28	0.519
	*osx*	1.01 ± 0.17^ab^	1.20 ± 0.23^b^	0.67 ± 0.19^a^	0.67 ± 0.22^a^	0.75 ± 0.19^ab^	0.66 ± 0.29^a^	1.12 ± 0.46^ab^	0.002	0.122	0.185	0.043
	*alp*	1.03 ± 0.26	1.12 ± 0.33	1.05 ± 0.38	1.37 ± 0.47	1.20 ± 0.24	1.77 ± 0.81	1.68 ± 0.55	0.054	0.141	0.555	0.529
	*oc*	1.06 ± 0.43	1.71 ± 0.46	0.98 ± 0.61	1.50 ± 0.82	1.04 ± 0.60	0.78 ± 0.50	0.79 ± 0.51	0.149	0.058	0.002	0.284
	*mgp*	1.03 ± 0.29^abc^	1.33 ± 0.50^c^	0.81 ± 0.24^ab^	0.83 ± 0.27^ab^	0.85 ± 0.19^ab^	0.74 ± 0.18^a^	1.22 ± 0.54^bc^	0.037	0.054	0.059	0.039
	*casr*	1.03 ± 0.27	1.21 ± 0.75	0.70 ± 0.29	1.20 ± 0.53	0.98 ± 0.21	0.99 ± 0.23	1.40 ± 0.48	0.215	0.667	0.662	0.175
	*pthr1*	1.00 ± 0.12^b^	0.84 ± 0.25^ab^	0.49 ± 0.26^a^	0.63 ± 0.21^ab^	0.48 ± 0.12^a^	0.64 ± 0.38^ab^	0.51 ± 0.25^a^	0.014	0.694	0.103	0.691
	*pthrp*	1.02 ± 0.19^ab^	1.64 ± 0.50^b^	0.78 ± 0.20^a^	1.61 ± 0.31^b^	1.00 ± 0.27^ab^	1.13 ± 0.58^ab^	1.18 ± 0.23^ab^	0.002	0.531	0.002	0.053

^∗^Different letters in a row denote significant differences between groups fed different diets (mean ± SD, *n* = 3, *p* < 0.05).

**Table 7:** Comparison of bone biomarkers and calcium regulator gene expression in gilthead seabream larvae between two sampling points day 7 (29 dph) and day 21 (43 dph) fed diets supplemented with several levels of vitamin D_3_ and vitamin K_3_.

Gene	Two-way ANOVA (*p* value)		
	Group	Day	Group ^∗^ day
*bmp2*	0.819	0.114	0.811
*runx2*	0.995	0.404	0.956
*osx*	0.043	0.368	0.918
*alp*	0.812	0.694	0.193
*oc*	0.401	0.912	0.985
*mgp*	0.429	0.461	0.947
*casr*	0.707	0.293	0.836
*pthr1*	0.839	0.095	0.749
*pthrp*	0.77	0.399	0.880

**Table 8:** Frequency of skeletal anomalies in gilthead seabream larvae fed diets supplemented with several levels of vitamin D_3_ and vitamin K_3_ for 21 days.

Skeletal anomalies (%)	0	0.06/70	0.06/170	0.13/70	0.13/170	0.40/70	0.40/170	One-way ANOVA (*p* value)	Two-way ANOVA (*p* value)		
									VD	VK	VD^∗^VK
Abdominal kyphosis	21.03 ± 20.08	24.94 ± 2.84	23.78 ± 6.66	15.22 ± 8.63	16.41 ± 12.45	19.57 ± 10.66	13.03 ± 7.94	0.810	0.353	0.684	0.828
Haemal lordosis	0.83 ± 1.44	5.13 ± 5.13	0.85 ± 1.48	2.24 ± 2.07	2.30 ± 2.19	0.83 ± 1.44	0.65 ± 1.13	0.332	0.307	0.231	0.267
Total anomalies	21.86 ± 21.28	30.07 ± 7.64	24.64 ± 6.69	17.46 ± 8.59	18.71 ± 12.77	20.40 ± 10.80	13.68 ± 9.00	0.731	0.287	0.527	0.826

## Data Availability

The data used to support the findings of this study are included in the article. However, more data could be available from the corresponding author upon request.
